# Harmonized Z-Scores Calculated from a Large-Scale Normal MRI Database to Evaluate Brain Atrophy in Neurodegenerative Disorders

**DOI:** 10.3390/jpm12101555

**Published:** 2022-09-21

**Authors:** Norihide Maikusa, Yoko Shigemoto, Emiko Chiba, Yukio Kimura, Hiroshi Matsuda, Noriko Sato

**Affiliations:** 1 Center for Evolutionary Cognitive Sciences, Graduate School of Art and Sciences, The University of Tokyo, Tokyo 113-8654, Japan; 2 Department of Radiology, National Center of Neurology and Psychiatry, Tokyo 187-8551, Japan

**Keywords:** Alzheimer’s disease, MRI, Z-score, harmonized, mild cognitive impairment

## Abstract

Alzheimer’s disease (AD), the most common type of dementia in elderly individuals, slowly and progressively diminishes the cognitive function. Mild cognitive impairment (MCI) is also a significant risk factor for the onset of AD. Magnetic resonance imaging (MRI) is widely used for the detection and understanding of the natural progression of AD and other neurodegenerative disorders. For proper assessment of these diseases, a reliable database of images from cognitively healthy participants is important. However, differences in magnetic field strength or the sex and age of participants between a normal database and an evaluation data set can affect the accuracy of the detection and evaluation of neurodegenerative disorders. We developed a brain segmentation procedure, based on 30 Japanese brain atlases, and suggest a harmonized Z-score to correct the differences in field strength and sex and age from a large data set (1235 cognitively healthy participants), including 1.5 T and 3 T T1-weighted brain images. We evaluated our harmonized Z-score for AD discriminative power and classification accuracy between stable MCI and progressive MCI. Our procedure can perform brain segmentation in approximately 30 min. The harmonized Z-score of the hippocampus achieved high accuracy (AUC = 0.96) for AD detection and moderate accuracy (AUC = 0.70) to classify stable or progressive MCI. These results show that our method can detect AD with high accuracy and high generalization capability. Moreover, it may discriminate between stable and progressive MCI. Our study has some limitations: the age groups in the 1.5 T data set and 3 T data set are significantly different. In this study, we focused on AD, which is primarily a disease of elderly patients. For other diseases in different age groups, the harmonized Z-score needs to be recalculated using different data sets.

## 1. Introduction

Alzheimer’s disease (AD) is the most common cause of dementia, and it typically starts with memory impairment at the earliest clinical stage. Mild cognitive impairment (MCI), a less severe condition than AD, increases the risk of developing AD.

Magnetic resonance imaging (MRI) is an excellent biomarker to quantify AD progression. The use of MRI in the morphometric or volumetric measurement of brain atrophy, evaluating parameters such as cortical thickness, hippocampus volume, and whole-brain volume, improved the diagnostic process. These measurements can also be used to assess the effectiveness of any applied therapies.

Several established methods exist to analyze cortical and subcortical volume and to use cortical volume from MRI data as a surrogate biomarker for AD and MCI [1,2,3,4,5,6,7,8,9,10,11,12,13,14,15]. Especially, a volume measurement pipeline called FreeSurfer [1,2] has been widely used for the volume and thickness measurement of anatomical regions of interest (ROI) in brain imaging clinical research.

Thus, brain atrophy is an important surrogate biomarker for AD. However, brain atrophy also occurs with age in cognitively normal participants; therefore, it is necessary to use an index that considers typical age-related atrophy.

Recently, multi-site clinical studies have been expanding worldwide to elucidate AD mechanisms and establish a useful surrogate biomarker for AD. Some of these studies are the Open Access Series of Imaging Study (OASIS) [16], The Australian Imaging, Biomarker & Lifestyle Flagship Study of Ageing (AIBL) [17], the Alzheimer’s Disease Neuroimaging Initiative (ADNI) [18], and the Japanese Alzheimer’s Disease Neuroimaging Initiative (J-ADNI) [19]. However, measurement bias in MRI data from different scanners has been reported, and all brain image analysis methods are affected by this bias. MRI data harmonization is an essential process for multi-site imaging studies to ensure the reliability of statistical analysis and reduce non-biological bias [20,21,22].

In this study, we used 30 Japanese brain atlases subdivided into 131 anatomical regions for the fast and highly accurate segmentation and brain analysis to calculate brain structures volume. We segmented 1235 normal control (NC) participants (1089 scanned by 3 T MRI and 146 by 1.5 T MRI).

We propose a harmonized Z-score for each anatomical ROI from the NC group as a universal reference for brain atrophy, independent of age, gender, and magnetic field strength. In the harmonized Z-score, we consider several confounding factors (i.e., sex, scanner field strength, 1.5 T or 3 T, estimated total intracranial volume (eTIV), and age). After harmonization of the brain structures volume, we can minimize the difference between subgroups for each covariate (i.e., males and females, 1.5 T and 3 T MRI scanner) and evaluate brain atrophy correcting for the effects of age and whole-brain volume.

## 2. Materials and Methods

### 2.1. MRI Acquisition

The three-dimensional (3D) T1-weighted images of the NC data set were obtained from two different protocols on 3 T MRI scanners at the National Center of Neurology and Psychiatry: 693 individuals underwent Protocol 1, and the other 438 individuals underwent Protocol 2. On the other hand, all the AD and MCI patients underwent Protocol 1. Protocol 1: on 3 T MR system (Philips Medical Systems, Best, The Netherlands): repetition time (TR)/echo time (TE), 7.18 ms/3.46 ms; flip angle, 10 degrees; number of excitations (NEX), 1; 0.68 × 0.68 mm ${}^{2}$ in plane resolution; 0.6 mm effective slice thickness with no gap; 300 slices; matrix, 384 × 384; field of view (FOV), 261 × 261 mm. Protocol 2: 3 T MR system (Verio, Siemens, Erlangen, Germany): TR/TE, 1800 ms/2.25 ms; flip angle, 9 degrees; NEX, 1; 0.87 × 0.78 mm ${}^{2}$ in plane resolution; 0.8 mm effective slice thickness with no gap; 224 slices; matrix, 320 × 280; FOV, 250 × 250 mm. All data were collected after obtaining informed consent from participants, and all methods were carried out in accordance with relevant guidelines and regulations. This study was approved by the Institutional Review Board at the National Center of Neurology and Psychiatry (project identification code: A2020-001, date of approval: 23 Marth 2020), Tokyo, Japan.

We also obtained 1.5 T T1-weighted MR images from the Japanese Alzheimer’s Disease Neuroimaging Initiative (J-ADNI) data set, which were provided by the National Bioscience Database Center in Japan (https://humandbs.biosciencedbc.jp/hum0043-v1, accessed on 22 August 2022). Data were acquired using 1.5 T MRI scanners (GE Healthcare, Siemens and Philips) and preprocessed with non-parametric non-uniform normalization (N3) [23] and phantom-based distortion correction [24]. We divided the total of 507 participants into four groups—NC, AD, stable MCI (sMCI), and progressive MCI (pMCI)—based on the following criteria:

NC participants: Mini-Mental State Examination (MMSE) score 24–30, Clinical Dementia Rating (CDR) Scale 0, non-depressed, no memory complaint.
AD patients:   MMSE score 20–26, CDR 0.5 or 1, and memory complaints.
sMCI patients:  MMSE score 24–30, memory complaints (preferably corroborated by an informant), objective memory loss measured, CDR 0.5, absence of significant levels of impairment in other cognitive domains, essentially preserved activities of daily living, diagnosis of MCI for ≥36 months.
pMCI patients:  MMSE score 24–30, memory complaints (preferably corroborated by an informant), objective memory loss measured, CDR 0.5, absence of significant levels of impairment in other cognitive domains, essentially preserved activities of daily living, diagnosis of MCI at baseline and conversion to AD within 6–36 months.

All the J-ADNI participants considered had taken the MMSE, CDR, Alzheimer’s Disease Assessment Scale (ADAS), and Functional Activity Questionnaire (FAQ) as neuropsychological screening tools. The evaluating MRI data were acquired at the baseline.

Table 1 summarizes the demographics of the participants.

### 2.2. Image Pre-Processing

All MR images were corrected for intensity inhomogeneity using the B1 correction algorithm [25] and a non-parametric non-uniformity intensity normalization (N3) algorithm [23]. Subsequently, phantom-based distortion correction [24] was performed to normalize variations between MRI scanners. Subsequently, we used the Computational Anatomy Toolbox (CAT12) based on SPM12 for image segmentation of T1-weighted MR images into three types of brain tissues (GM, WM, and CSF) and background. It is assumed that the histogram of image intensity follows a Gaussian mixture model. Accordingly, the existing possibilities of the three tissue types can be calculated for the image intensity at an arbitrary voxel, p(T|I), using the Bayesian estimation.

### 2.3. Segmentation and Calculation of Brain Structures Volume by Multi-Atlas Fusion

We used a segmentation procedure that incorporates a joint-label fusion method [26,27] and corrective learning [28] with an automatic selection of five among thirty atlases. Each atlas included 131 manually traced ROIs based on T1-weighted images. Briefly, the segmentation procedure involves the following algorithms: (1) The target T1-weighted brain image was divided into 30 large regions by non-linear warping from the large-regions atlas in MNI space (described by the Montreal Neurological Institute) to the target T1-weighted image. The large-regions atlas was created by grouping some ROIs into large regions in the “neuromorphometrics atlas” attached in the CAT (https://neuro-jena.github.io/cat/, accessed on 22 August 2022), based on prior anatomical knowledge. (2) Five atlases were automatically selected from 30 manually traced atlases, according to the Pearson correlation between the 30 atlases and the target image for each large region. The Mmanually traced atlases were created by one Ph.d.PhD researcher outside our researchexternal to our group, who has been involved in brain imaging research. The qualities quality of manually tracedthese atlases were checked was assessed by a radiologist (H.M. ) in our study group. (3) We created a diffeomorphic anatomical registration through exponentiated lie algebra (DARTEL) template from the five selected atlases and the target image at each large region. Subsequently, the five atlases were warped to the target image via the large-region DARTEL templates. (4) Using the joint label fusion (JLF) method and SegAdapter, the target image was segmented into 133 ROIs from the fused selected atlases at each large region. (5) The volumes of the ROIs are calculated after considering the partial volume effects by the posterior probability maps based on the CAT12 toolbox. Since the segmentation procedure can be performed with parallel processing for each large region, the processing time was reduced.

The segmentation procedure was executed using a multi-core computer system (OS: CentOS 7.2, CPU: Intel Xeon E5-2600 24 cores, memory: 96 GB), and the processing time was measured.

### 2.4. Calculating Process for the Harmonized Z-Score

According to the report of Ma et al. [22], we can model and harmonize the covariates of no interest (i.e., age, field strength, eTIV, and sex) by a general linear model. In this study, we focused on the magnetic field strength as the main factor that describes the characteristics of MRI scanners. Ideally, the MRI scanner model and acquisition parameters should also be considered; however, but it is not realistic to considerinclude all of these including variables, which may change newly manufactured in the future, when consideringfor clinical applications. SoTherefore, we only considered the field strength, which is the most influential factor in volumetric measurements to increase versatility by using a simpler model. We defined the structure volume and all the other covariates as follows:

(1) $$\begin{array}{c} log_{10}V_{i,j}=\beta_{0,j}+\beta X_{i}+\epsilon_{i,j} \end{array}$$

where $V_{i,j}$ is the structural volume for the *i*-th subject and j-th ROI, and $X_{i}$ is the design matrix of the covariates, i.e., age, field strength (0: 3 T, 1: 1.5 T), gender (0: female, 1: male) and $log_{10}$(eTIV). After removing the effect of the covariates, we can calculate the harmonized Z-score at each ROIs as:

(2) $$\begin{array}{c} Z_{i,j}=\epsilon_{i,j}/\sqrt{\left(1+\frac{1}{N}+\frac{{\left(X_{i}-\bar{X}\right)}^{T}\sum {}^{-1}\left(X_{i}-\bar{X}\right)}{N-1}\right){\hat{\sigma_{j}}}^{2}} \end{array}$$

where $\hat{\sigma_{j}^{2}}$ is the unbiased distribution, *N* is the number of participants, and ∑ is the covariance matrix of participants.

We calculated the harmonized Z-score for each ROI from 1089 NC participants scanned by 3 T MRI at NCNP and 146 NC participants scanned by 1.5 T MRI from the J-ADNI data set. Moreover, we evaluated and excluded the outliers of Z-scores using the Smirnov–Grubbs test with a 5% significance level; then, we recalculated the Z-scores according to the same process on Python 3.6.6.

In this article, we selected and presented some covariate combinations to illustrate scatter plots of age correlations: (a) no harmonization (raw variable); (b) field strength only; (c) the combination of field strength and eTIV; (d) the combination of field strength, eTIV, and sex. Therefore, we calculated the efficacy of the harmonized Z-score to distinguish groups using four different models: (a) age only; (b) the combination of age and field strength; (c) the combination of age, field strength, and eTIV; (d) the combination of age, field strength, eTIV, and sex.

### 2.5. Evaluation of the Harmonization of Different Field Strength and Sex

We used the Kolmogorov–Smirnov test to measure the separation of the Z-score distributions and quantitatively compare the separation of the sample distributionres, thus comparing the Z-scores between NC images from 1.5 T and 3 T scanners. If the harmonization works properly, the difference between the Z-score distribution in subgroups with different covariates (i.e., field strength and sex) in NC participants will be smaller than the non-harmonized difference.

### 2.6. Evaluation of Harmonized Z-Score for AD Detection and Classification of sMCI/pMCI

Finally, we evaluated the accuracy of the harmonized Z-score in the ROIs to detect differences between the AD and NC groups, and the sMCI and pMCI groups. The harmonized Z-score was analyzed using the receiver operating characteristic (ROC) curves and the area under the curve (AUC). The ROC curves of each ROI were drawn based on the trade-off between sensitivity and specificity for discriminating between diagnostic groups. A higher AUC indicates higher sensitivity and specificity using the harmonized Z-score in the ROIs.

## 3. Results

We performed the segmentation procedure on all T1-weighted brain images; most of the processing procedures were completed within 30 min.

### 3.1. Evaluation of the Harmonization of Different Field Strength and Sex

**Figure 1:**
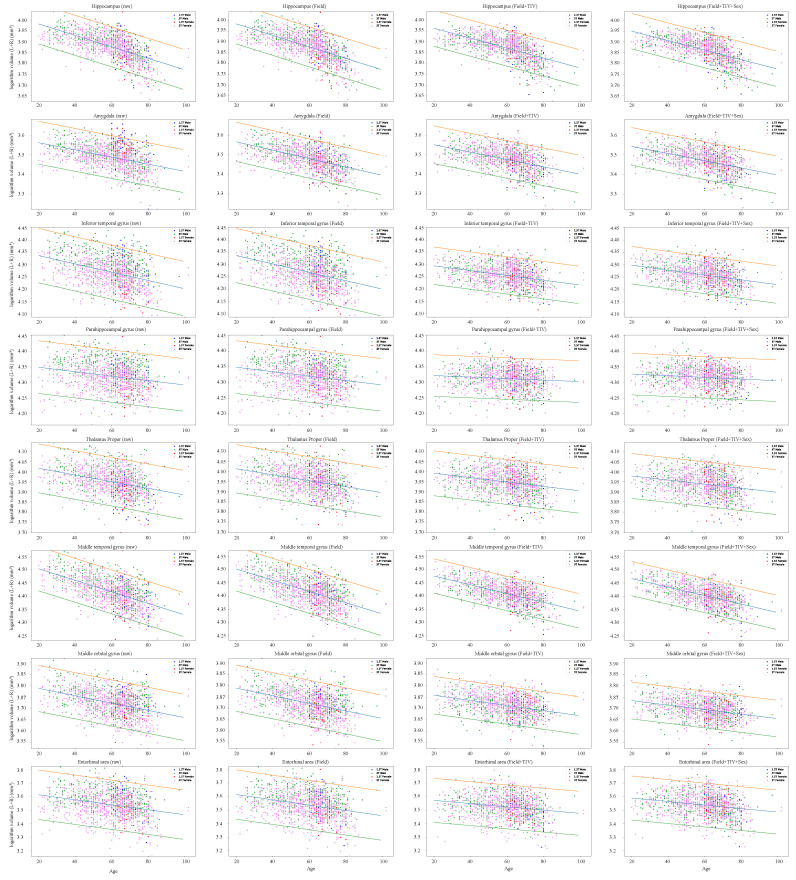
Correlation between age and some selected structures.

Figure 1 shows the correlation between raw and harmonized volumes of a set of eight ROIs associated with cognitive function for the NC group. The male/female and 1.5 T/3 T data are shown overlapped.

Correlation between the age (*x* axis) and some selected structure logarithm volumes (*y* axis) was shown for the NC group for 1.5 T males (blue), 3 T males (green), 1.5 T females (violet) and 3 T females (red).

The blue line represents the regression line for age, and the red and green lines are the 95% confidence intervals. An overall negative correlation between age and ROI volumes within the gray matter was found. The coefficients for the harmonized Z-score calculation in all ROIs are listed in Additional file 1.

The 95% confidence intervals became narrower after each harmonization and improved particularly when the magnetic field strength and eTIV or all covariates were corrected. To quantitatively assess the shift of the kernel density estimate function (KDEF) of the Z-score before and after harmonization, accounting for each covariate, we performed the K-S test between the 1.5 T NC and 3 T NC groups. Before harmonization, in 72/133 ROIs, the null hypothesis (i.e., the two structural volume distributions scanned by 1.5 T MRI and 3 T MRI came from the same population) was rejected. However, it was correctly not rejected after harmonization.

**Figure 2:**
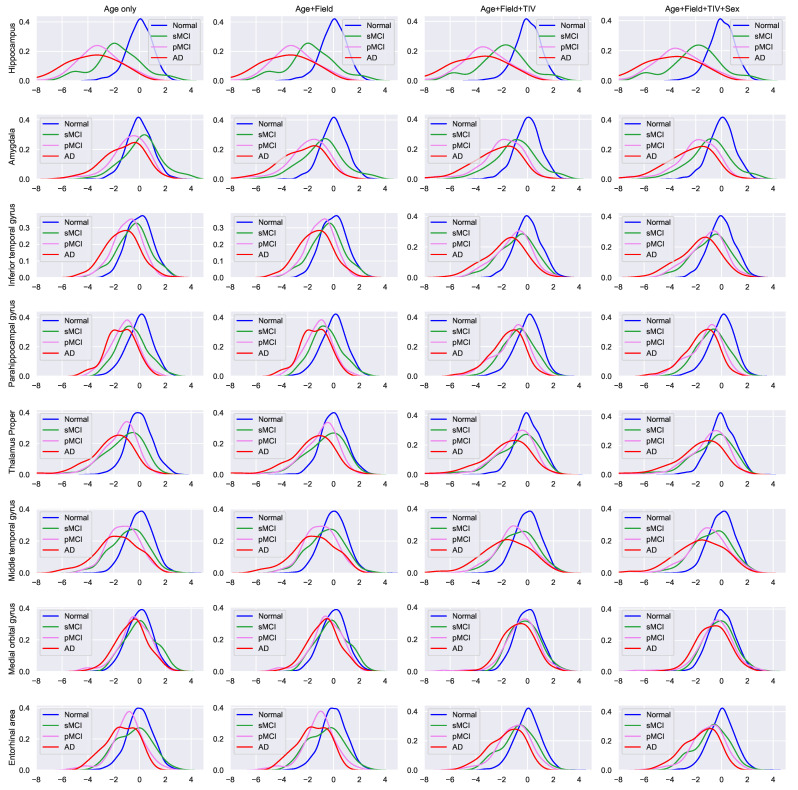
The kernel density estimation function of the harmonized Z-score taken from a select few gray matter structures. Blue line: NC, green line: sMCI, violet line: pMCI, red line: AD.

Figure 2 shows the KDEFs of the harmonized Z-scores in selected structures. The KDEFs of the Z-scores showed the brain atrophy of AD on these structures compared to the NC group. When the Z-score shows a negative value, it indicates that the ROI of participants is atrophied compared to the NC, significantly if the Z-score is lower than −2.

After harmonization, the distribution of Z-scores in the AD and pMCI groups was shifted leftward in the hippocampus, amygdala, inferior temporal gyrus, parahippocampal gyrus, and middle temporal gyrus. These results show that the AD detection power using the Z-score is improved after harmonization including all covariates (field strength, eTIV, and sex). The statistical parameters for calculating the harmonized Z-score from each brain ROIs are showed in Table S1.

### 3.2. Discriminative Power between NC vs. AD and sMCI vs. pMCI

The AUC values of the comparisons of AD vs. NC and pMCI vs. sMCI are shown in Table 2. The results showed that the Z-score of the hippocampus has high AUC values: 0.96 for the right hippocampus, and 0.95 for the left.

## 4. Discussion

We analyzed 131 brain structural volumes in 1235 (3 T = 1089, 1.5 T = 146) cognitively normal participants, and we calculated the harmonized Z-score for each region considering age, field strength, eTIV, and sex. The harmonization of these covariates improved the reliability of assessing age-related atrophy in the normal database because the amplitude of the confidence interval decreased after harmonization. Quantitative evaluation based on the Kolmogorov–Smirnov test showed that the null hypothesis of the two structural volume distributions (1.5 T MRI and 3 T MRI) coming from the same population was not rejected. These results showed that harmonization is effective.

Our method achieved high AUC values (0.96) in the hippocampus to separate NC and AD and moderate AUC values (0.70) discriminating between pMCI and sMCI. Elahifasaee et al. showed that a pMCI/sMCI classification accuracy of 65.94% could be achieved based on feature decomposition and kernel discriminant analysis [29]. Several studies have shown that discrimination between sMCI and pMCI is a difficult task. Our results cannot compare directly to Farzaneh’s results because the metrics are different; however, since our results are based only on the univariate Z-score, our results showed a reliable accuracy to classify sMCI and pMCI in comparison with previous studies.

There are some limitations to our study. The measurement bias may include the MRI manufacturer, image acquisition protocol, scanner coil, and field strength; we only considered field strength in our study. Moreover, there are innumerable possible combinations of other covariates, and we tried to increase the generality in clinical applications by considering a simple model.

## 5. Conclusions

Our results showed that our method could be effective for AD detection, with high accuracy. Moreover, it can be used for sMCI/pMCI discrimination without being affected by the different field strengths, thus having high generalization capability. Most of our database, in fact, consisted of participants scanned by 3 T MRI, while the evaluation data set comprised patients all scanned by 1.5 T MRI. Furthermore, our method can be expected to improve the accuracy of the multivariate Z-score approaches, such as machine learning and other multivariate analyses. We believe that multivariate Z-scores derived from whole-brain ROIs can be applied to the type classification of dementia and may be a useful biomarker of other neurodegenerative disorders.

## Figures and Tables

**Table 1:** Characteristics of the study participants.

	Normal Data Base		Evaluation Data Set			
**Source**	**NCNP**	**J-ADNI**		**J-ADNI**		
**Filed Strength**	**3 T**	**1.5 T**		**1.5 T**		
Category	NC	NC		sMCI	pMCI	AD
Number of participants	1089	146		102	112	147
Mean Age (SD)	58.5 (14.3)	67.6 (5.66)		72.8 (6.10)	73.0 (5.54)	73.5 (6.60)
Male (Female)	375 (714)	68 (78)		57 (45)	47 (65)	63 (84)

**Table 2:** The area showed the AUC value of 80 or more for NC vs. AD and an AUC value of 60 or more for sMCI vs. pMCI.

AD vs. NC			pMCI vs. sMCI		
**Region**	**AUC**	**Balanced Accuracy**	**Region**	**AUC**	**Balanced Accuracy**
Right Hippocampus	0.96	0.88	Right Hippocampus	0.70	0.68
Left Hippocampus	0.95	0.89	Left Hippocampus	0.68	0.67
Right Amygdala	0.88	0.80	Left Amygdala	0.66	0.63
Left Amygdala	0.86	0.79	Right Amygdala	0.64	0.61
Right ITG	0.82	0.75	Right Entorhinal area	0.60	0.62
Left Thalamus Proper	0.80	0.74	Left PHG	0.60	0.60
Left PHG	0.80	0.76	Right MOrG	0.60	0.59
Right PHG	0.80	0.74			
Right MTG	0.80	0.74			

PHG, Para Hippocampal Gyrus. MTG, Middle Temporal Gyrus. ITG, Inferior Temporal Gyrus. MOrG, Medial Orbital Gyrus.

## Data Availability

The datasets used and/or analyzed during the current study are available from the corresponding author on reasonable request.

## Supplementary Materials

The following supporting information can be downloaded at: https://www.mdpi.com/article/10.3390/jpm12101555/s1, Table S1: The coefficients for harmonized Z-score calculation in all ROIs.

## Abbreviations

The following abbreviations are used in this manuscript:

AD	Alzheimer’s Disease
MCI	Mild Cognitive Impairment
sMCI	Stable Mild Cognitive Impairment
pMCI	Progressive Mild Cognitive Impairment
NC	Normal Control
PHG	Para Hippocampal Gyrus
ITG	Inferior Temporal Gyrus
MTG	Middle Temporal Gyrus
MOrg	Medial Orbital Gyrus
